# Experimental Observation of Linear and Rotational Doppler Shifts from Several Designer Surfaces

**DOI:** 10.1038/s41598-019-45516-1

**Published:** 2019-06-20

**Authors:** Baiyang Liu, Hongchen Chu, Henry Giddens, Ronglin Li, Yang Hao

**Affiliations:** 1 Queen Mary University of London, School of Electronics Engineering and Computer Science, London, E1 4NS UK; 2 South China University of Technology, School of Electronics and Information Engineering, Guangzhou, 510640 China; 3 Soochow University, College of Physics, Optoelectronics and Energy and Collaborative Innovation Center of Suzhou Nano Science and Technology, Suzhou, 215006 China

**Keywords:** Micro-optics, Electrical and electronic engineering

## Abstract

An orbital angular momentum (OAM) carrying beam has the ability to detect a spinning surface from its rotational Doppler effect. However, a mixture of linear and rotational Doppler effects can occur when an OAM beam is illuminated to a target, with not only spins but also vibrations. In this paper, we experimentally observe using OAM carrying beams, both linear and rotational Doppler effects from several designer surfaces. Specifically, a spinning polarization-independent metasurface, helicoidal reflector and propeller are applied respectively in this study. We demonstrate by the use of two microwave beams with opposite OAM to separate rotational Doppler shift from micro-Doppler shift. The proposed method can also be applied to measure the spinning speed of rotational objects, which have wider applications in intelligent sensing, radar and quantum optics.

## Introduction

It is very well known that, an electromagnetic (EM) system can radiate not only linear momentum but also angular momentum^1^. The angular momentum consists of spin and orbital part. Spin angular momentum (SAM) and orbital angular momentum (OAM) are associated with the polarization and phase of the field, respectively^2–5^. A general characteristic of an OAM beam is that the field has a helical transverse phase structure exp(*ilϕ*), in which *ϕ* is the transverse azimuthal angle and *l* is an unbounded integer representing for the OAM state number. An OAM beam with helical phase front can be obtained by introducing azimuthal phase dependence exp(*ilϕ*) into the beam. In optics, the generation of OAM beams is achieved through the use of spiral phase plates^6–10^ which are able to modulate the length of optical path to change the phase delay. Given the different frequency range from optics, a uniform circular antenna array with successively phase shift is commonly used for OAM generation at radio frequency^11–14^. A helicoidal reflector is also proposed to generate OAM beam at radio frequency^15,16^. In addition, phase gradient metasurfaces have attracted more and more attention due to their unique ability to flexibly manipulate the wavefront of electromagnetic waves. In this way, metasurfaces covering the phase change range from 0 to 2*π* can be designed as an OAM generator^17–20^.

The orthogonality of OAM beams^5^ has recently found its practical use in communication systems, one can therefore establish a line-of-sight communication link with different OAM beams carrying independent data at the same frequency to increase the channel capacity^21–28^. OAM multiplexing is not restricted to optical domain, there is great interest to also apply OAM multiplexing at radio frequency and millimeter-wave communication systems to increase spectral efficiency and channel capacity^23,29^. Moreover, an infinite number of states have been mapped to different digital symbols for wireless data encoding and decoding^30,31^.

Furthermore, OAM beams have also been demonstrated to detect a spinning object via the rotational Doppler effect^32^. The Doppler shift is a well-known phenomenon in which the relative velocity, *v*, between a wave and an observer causes a frequency shift, Δ*f*, of the wave. However, the rotational Doppler effect of OAM waves is less well-known^32–34^. In an OAM beam, the Poynting vector has an azimuthal component at every position within the beam. The rotational Doppler effect is generated by the azimuthal component of the Poynting vector and relative rotational motion^35–37^. The rotation of a linearly polarized beam carrying OAM results in a frequency shift of *l*Ω*/*2*π*, where Ω is the angular frequency of rotation between source and observer and *l* is the OAM mode of the beam. The rotational Doppler shift due to the spin and orbital angular momentum components of the beam act in an additive way. For a circularly polarized OAM beam, the rotational Doppler shift is (*l* + *σ*)Ω*/*2*π*, where *σ* = ± 1 is expressed as left or right circular polarization, which has been demonstrated by spin-orbital coupling of light at spinning metasurfaces^38^.

The rotational Doppler effect of an OAM beam has been recently used to detect the rotational motion of an object and molecule spinning^35,39^. Rotational Doppler shift can even detect both rotation speed and the rotational symmetry of objects at photon-count level^40^. Moreover, if the target has a translational motion or a vibration in addition to their rotation, it might induce both linear and rotational Doppler effects^41^, in this case, by applying the OAM beam alone will not be able to measure the spinning speed of the target from the rotational Doppler effect due to an additional frequency shift modulated with the rotational frequency shift^42^. In this paper we demonstrate, through the use of a spinning polarization-independent metasurface, the ability to detect the rotational Doppler effect from a COTS radar system. The proposed metasurface with ring shape unit cells is polarization independent^43,44^ and can generate an OAM beam by reflecting an arbitrary polarized incident wave. In our demonstration, a radar transmits a normal electromagnetic wave illuminating on the proposed spinning metasurface, the wave interacts with the proposed metasurface and returns as an OAM wave with rotational Doppler shift^45,46^. As shown from spectrograms based on measured data, the frequency shift is determined by the angular velocity and OAM mode of the proposed metasurface. To further verify our hypothesis, a helicoidal reflector and a propeller of similar configuration have been used to study the detection of translational motions as well as rotations, in addition to those from an OAM metasurface. On one hand, the Poynting vectors of a wave reflected by a spinning helicoidal reflector have a skew angle with the beam axis that introduces rotational Doppler effect. On the other hand, the spinning helicoidal reflector has a continuous translational velocity that generates linear Doppler effect. The spectrogram in our experiment shows that the reflected wave by a helicoidal reflector undergoes multiple Doppler effects. Finally, in order to measure the spinning speed of a propeller, we use two opposite values of OAM beams to measure the frequency shift difference in order to obtain its spinning speed. The proposed method has the potential to measure the spinning speed of a rotational target with translational motion or vibration and therefore much wider applicability in many subject areas ranging from microwave to optics than what is demonstrated in this paper.

## Results

### Linear and rotational doppler effects

**Figure 1:**
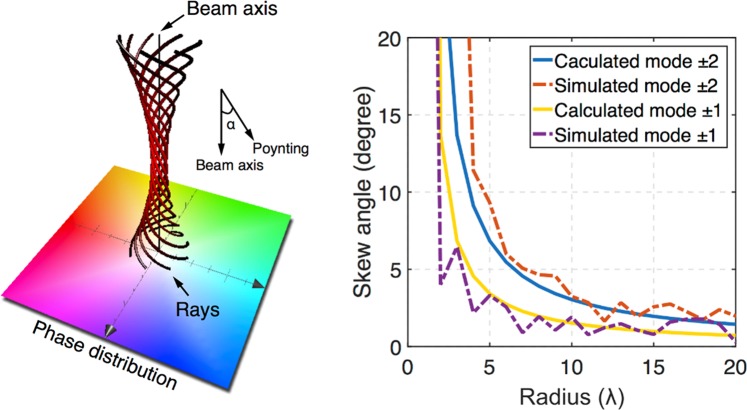
Skew angle of an OAM beam. (**a**) shows the rays and phase distribution of an OAM beam, the rays show an angle between the Poynting vector and beam axis. (**b**) shows the calculated and simulated skew angles. For *l* > 0, the OAM beam has a clockwise skew angle; for *l* < 0, the OAM beam has a counterclockwise skew angle. Two OAM beams of opposite values of *l* have the same absolute value of skew angle |*α*|. Here we calculate and simulate the skew angles |*α*| of *l* = ± 1 and *l* = ± 2 OAM beams.

In an OAM beam with mode denoted by *l*, which has a helical phase front and the rays are helixes, the angle *α* between the Poynting vector and beam axis is:

1 $$\alpha =\frac{l\lambda }{2\pi r}$$

where *λ* is the wavelength of the beam and *r* is the radius from the beam axis^32^. Therefore the OAM beam has an azimuthal momentum at every position within the beam. Figure 1(a) is the rays and phase distributions of an OAM beam, the rays are helixes and have a skew angle between the Poynting vector and beam axis. Here we calculate the skew angles of different OAM beams by Eq. (1) and simulate the skew angles, as shown in Fig. 1(b). The simulated OAM beams are generated by a circular phased array^2^. The simulation and calculated skew angles have a good agreement, confirming that the OAM beams from our simulation are carrying vortex momentum. The azimuthal momentum of OAM beam shown in Fig. 1 can generate the rotational Doppler effect.

**Figure 2:**
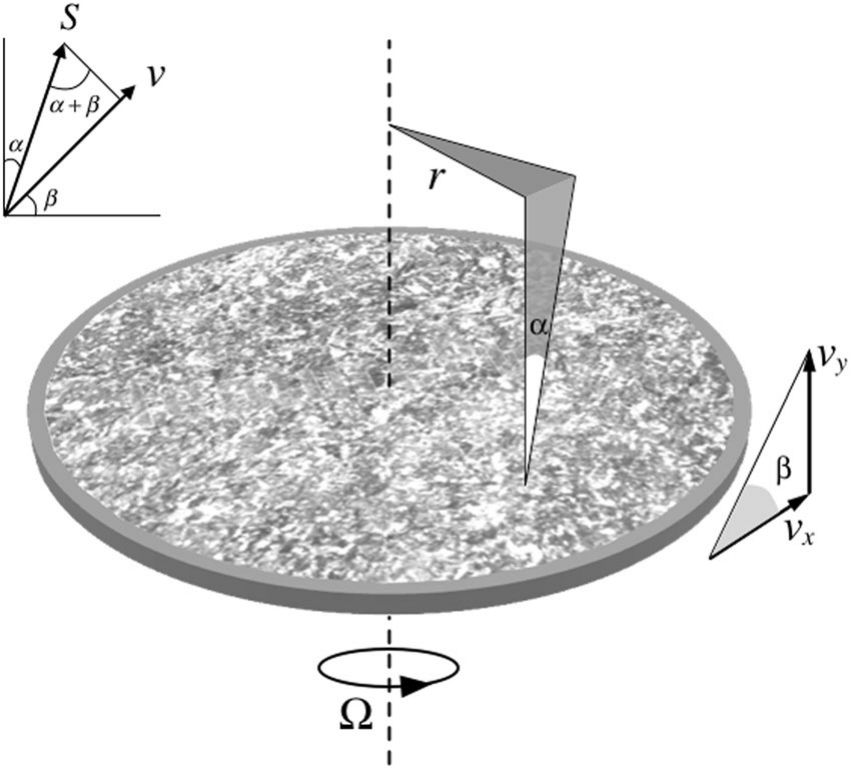
OAM beam scattered from a rough surface with a rotation and a translation motion causing multiple Doppler Effects.

For an OAM beam illuminating on a rough surface with not only a transversal rotation but also a longitudinal translation, as shown in Fig. 2, the reflected wave undergoes multiple Doppler effects. Let us denote *l* as the transmitted OAM mode, *r* is the radius from the beam axis, *α* is the skew angle (small *α*, sin*α* ≈ *α*, cos*α* ≈ 1), the horizontal velocity is *v*_*x*_ = Ω*r*, the vertical velocity is *v*_*y*_, the angle between the horizontal velocity (***v***_***x***_) and total velocity (***v***_***x***_ + ***v***_***y***_) is *β*, sin(*α* + *β*) is to calculate the vector projection of Poynting vector onto the velocity vector, *c* is the speed of light, *f*_0_ is the original frequency of the transmitted wave, and the frequency shift Δ*f* of the reflected wave can be calculated by^32^:

2 $${\rm{\Delta }}f=\,\sin (\alpha +\beta )\sqrt{{v}_{x}^{2}+{v}_{y}^{2}}\frac{{f}_{0}}{c}\approx (\alpha \,\cos \,\beta +\,\sin \,\beta )\sqrt{{v}_{x}^{2}+{v}_{y}^{2}}\frac{{f}_{0}}{c}=\frac{l{\rm{\Omega }}}{2\pi }+\frac{{v}_{y}{f}_{0}}{c}$$

Note that the first term is the rotational Doppler effect generated by the transversal rotation which is frequency independent and determined only by the OAM mode *l* and angular frequency Ω, and the second term is the linear Doppler effect generated by the longitudinal translation which is frequency dependent and related to the original frequency of the transmitted wave *f*_0_.

### Rotational doppler effect of spinning metasurface

**Figure 3:**
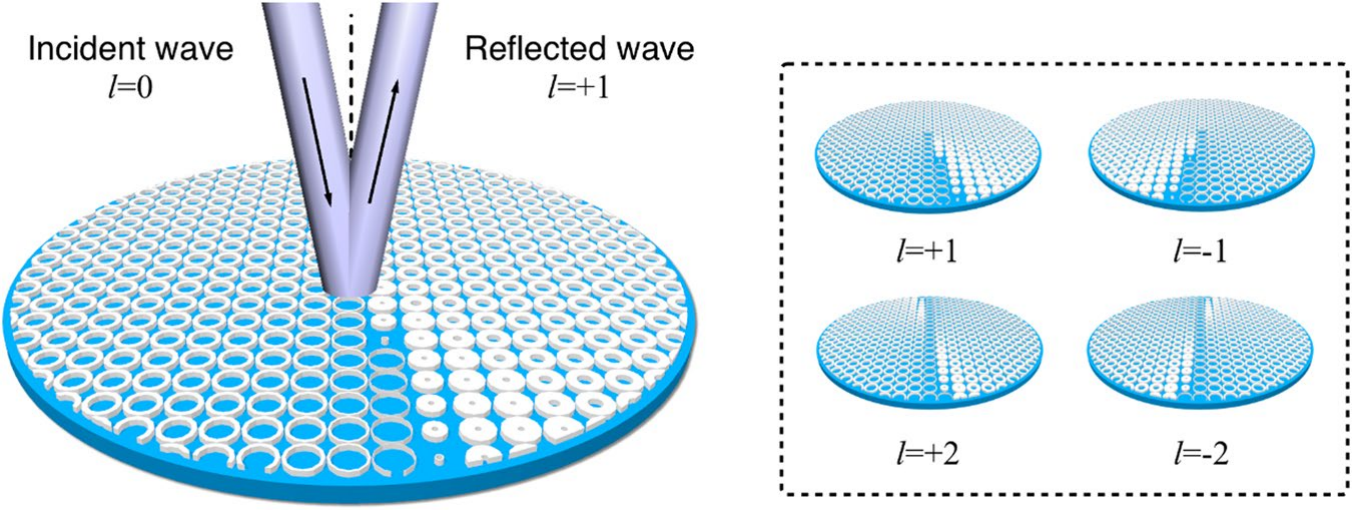
Polarization-independent OAM metasurfaces for rotational Doppler effect. Here we propose metasurfaces with ring shape unit cells to generate vortex beam with different OAM modes. The size of unit cell is *λ*/5 both in width and length. Varying the inner radius and outer radius of the unit we can obtain the reflection phase shift from 0 to 2*π* at the target frequency. A vortex beam can be obtained after a plane wave reflected by the proposed metasurfaces. Due to the polarization symmetrical structure of the metasurfaces, OAM beam can be generated while the metasurfaces are spinning. All of the metasurfaces (*l* = ± 1 and *l* = ± 2) are operating at 5.8 GHz.

For OAM generation, a helical phase and doughnut shape amplitude distribution are required. An OAM metasurface has a helical reflection phase, thus a normal wave reflected by an OAM metasurface can be transformed into an OAM beam^19,20^, while the metasurface is spinning, the transversal motion and the azimuthal momentum of the reflected OAM beam can generate rotational Doppler effect^38^. Here we propose to use a spinning metasurface to detect the rotational Doppler effect by a radar system. When the metasurface is spinning, the relative polarization state of the incident wave upon the metasurface is changing. In order to detect the rotational Doppler effect by a spinning metasurface, the metasurface should generate a helical phase beam for arbitrary polarization. Four different metasurfaces with ring shaped unit cells are designed in order to generate reflected *l*= ± 1 and *l* = ± 2 OAM beams. The metasurfaces have a polarization symmetrical structure and work for any polarizations, therefore the proposed metasurfaces can be used to generate OAM beam while spinning. The detailed information of the proposed metasurfaces is shown in the supporting information. Figure 3 shows the proposed metasurfaces used for the detection of the rotational Doppler effect.

**Figure 4:**
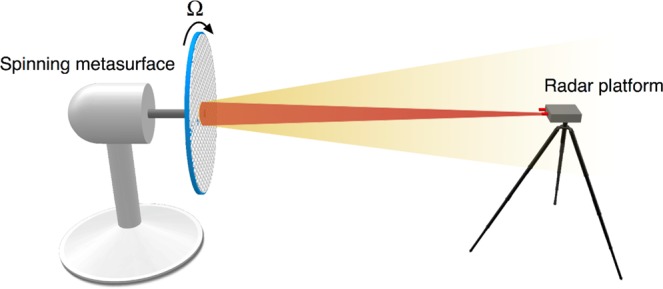
Spinning metasurface for rotational Doppler effect. The proposed polarization-independent OAM metasurface is rotated by a motor, the angular frequency of the metasurface is Ω = 23.87 × 2*π* rad/s. We measure the spectrograms of the received radar signal reflected by different metasurfaces with different OAM modes (*l* = ± 1 and *l* = ± 2).

A spectrogram is a visual representation of how the spectrum of electromagnetic waves varies with time. The spectrogram is defined as the square modulus of the short-time Fourier transform, and is a popular tool for time-frequency analysis^42^. In our demonstration, we rotate the proposed polarization-independent OAM metasurface by a motor. A radar transmits a signal and receives the scattered signal from the spinning OAM metasurface. The spectrogram is then measured to observe the frequency shift. The experiment setup is shown in Fig. 4. In this case, the frequency shift due to rotational Doppler effect can be calculated by

3 $${\rm{\Delta }}f=\frac{l{\rm{\Omega }}}{2{\pi }}$$

**Figure 5:**
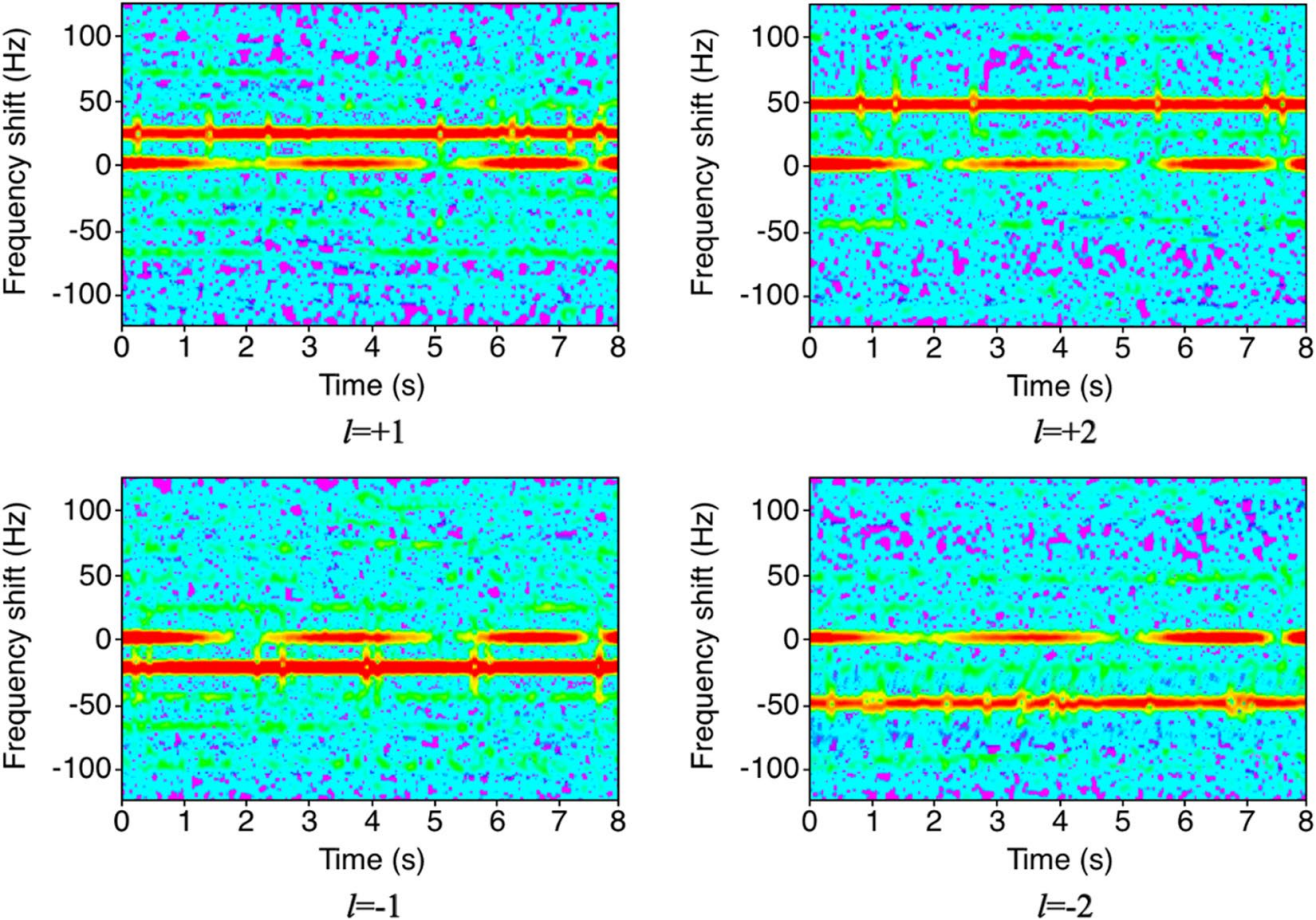
Measured spectrograms of reflected signal by different metasurfaces with different OAM modes. The frequency shift of *l* = + 1, *l* = −1, *l* = + 2 and *l* = −2 spinning metasurface is approximately +23 Hz, +48 Hz, −23 Hz and −48 Hz, respectively. The errors are less than 3.6%.

In Eq. (3), *l* is the OAM mode of the metasurface, Ω is the rotation of angular frequency of the spinning metasurface. Once the metasurface and radar have been aligned, the unwanted off-axis movement is easily avoided as in this case both the source and detector can be fixed in the same location with no movement. It should be noted that, if the metasurfaces and the radar are not perfectly aligned, the reflected wave may not be perfect OAM beam and the frequency shift may not be constant. The angular frequency of the spinning metasurfaces is Ω = 23.87 × 2*π* rad/s, which is measured by a high-speed camera, the high-speed camera records the spinning metasurface, from slow motion playback we can count the revolutions per second then calculate the spinning speed. We can therefore expect to detect a frequency shift of approximately ±24 Hz for OAM modes of *l* = ± 1, and approximately ±48 Hz for OAM modes with *l* = ± 2. Figure 5 shows the measured spectrograms of our experiment, the measurement distance is 1 meter. It should be note that, when the distance increases, the power of the OAM beam will drop faster than a normal beam thus it is more difficult to be detected by the radar^2^. The frequency shifts have a good agreement with the theoretical rotational Doppler shifts calculated by Eq. (3). The measured spectrogram at 0 Hz is not continuous because of the radar signal processing algorithm. The 0 Hz frequency shift is filtered when the power of the received signal is too low. Moreover, the low power harmonic frequency shifts in the spectrogram are on one hand due to other reflected wave from the ground, and on the other hand due to the metasurface, which does not continuously cover the full 2*π* reflection phase. The effect is that it behaves as a space-time-modulated reflector^47^.

**Figure 6:**
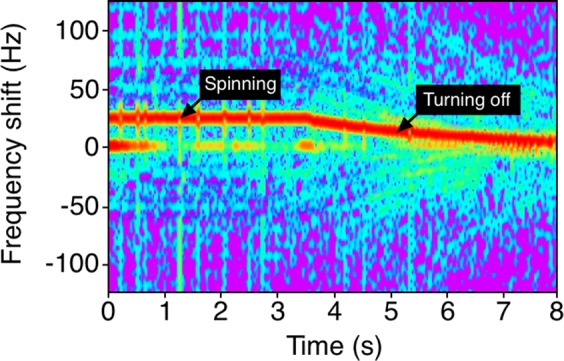
Measured spectrogram of reflected signal by *l* = + 1 metasurface from spinning to turning off. At the beginning, the *l* = + 1 metasurface has an angular frequency of Ω = 23.87 × 2*π* rad/s, the spectrogram shows an about +24 Hz frequency shift. After that we turn off the motor and the angular frequency of the metasurface is dropping down, at that period, the spectrogram shows the frequency shift is decreasing as well.

Moreover, we demonstrate the spectrogram of an *l* = + 1 metasurface from spinning to turning off, the spectrogram is shown in Fig. 6. From the spectrogram we can see that the influence of the angular frequency of the metasurface on the rotational Doppler shift. It is clearly noted that the rotational Doppler shift was reduced as the spinning speed of the metasurface decreased.

### Linear and rotational doppler effects of a helicoidal reflector and spinning speed detection of a propeller

**Figure 7:**
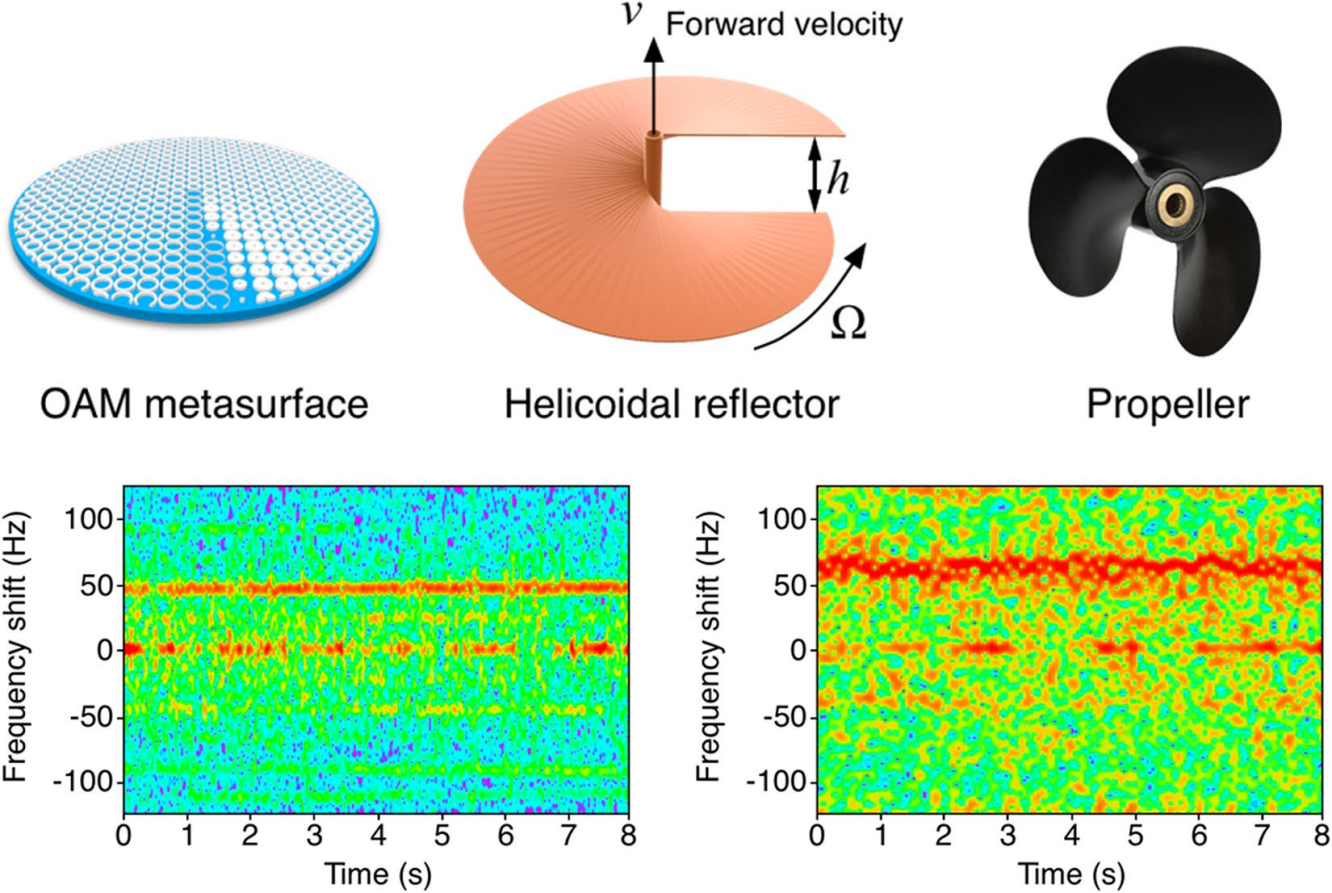
Muliple Doppler effects of an *l* = + 1 helicoidal reflector. (**a**) shows the structure of the proposed OAM metasurface. (**b**) shows the structure of an *l* = + 1 helicoidal reflector under test, (**c**) shows the structure of a three-blades propeller. The propeller has the similar structure as a helicoidal reflector. The spinning of the helicoidal reflector causes angular frequency Ω = 23.87 × 2*π* and forward velocity *v*. (**d**) is the measured spectrogram of a spinning *l* = + 1 helicoidal reflector by a radar operating at 5.8 GHz, the total frequency shift consists of rotational Doppler shift and linear Doppler shift. (**e**) is the measured spectrogram of a three-blades propeller.

A helicoidal reflector can be designed by elevating a surface at different azimuthal angles. The helicoidal reflector, or vortex reflector, can be used to generate an OAM beam due to the introduction of an azimuth phase shift of the reflected waves. From the reflection phase point of view, an OAM metasurface can be considered as a helicoidal reflector. Particularly, a propeller consists of three helicoidal blades which can also be used to generate beam with skew angle, the skew angle of the reflected waves and the transversal spinning of the propeller can generate rotational Doppler effect, as shown in Fig. 7. The propeller with spinning speed Ω = 23.87 × 2*π* rad/s on one hand can reflect the wave processing OAM causing the rotational Doppler effect. On the other hand the spinning of the propeller generates thrust, therefore a translational motion with a forward velocity *v* generates the Doppler effect. Figure 7 shows the spectrogram of a normal 5.8-GHz incident wave reflected from a spinning *l* = + 1 helicoidal reflector, the detailed information of the helicoidal reflector is shown in the supporting information, note that the frequency shift generates by both rotational and translational motion, because the total frequency shift is approximately 48 Hz is larger than the expected rotational Doppler shift which is 23.87 Hz, the rest of the frequency shift is generated by the forward speed. The elevation height *h* of the helicoidal reflector is *λ*/2, in a radar system, the forward speed *v* = 2*h*Ω/2*π* = 1.23 m/s. The frequency shift due to the forward speed can be calculated by *vf*_0_/*c* which is 23.87 Hz and is equal to the rotational Doppler shift for the helicoidal reflector. Therefore the total frequency shift is about 48 Hz, which has a good agreement with the measured spectrogram as shown in Fig. 7(d). Figure 7(e) shows the spectrogram of a spinning three-blades propeller, which has a similar pattern as the spectrogram of the helicoidal reflector. The imperfect spectrogram is due to the three-blades propeller is not a perfect helicoidal reflector. The elevation height of the three-blades propeller is 42 mm that can be consider as *l* ≈ + 1.6 OAM reflector at 5.8 GHz. However, due to the three-blades propeller does not have a perfect helicoidal reflector configuration, the measured frequency shift has a slight difference with the calculated result by Eq. (2).

The skew angle of the reflected wave and forward velocity are dependent on the elevation height of the propeller. Without knowing the structure of propeller, we cannot distinguish between the rotational Doppler shift and the linear Doppler shift, therefore we cannot measure the spinning speed of propeller by the total frequency shift. In order to detect the spinning speed of a propeller, here we propose to illuminate two OAM beams of opposite values, the frequency shift of the scatting waves have the same Doppler shift but opposite value of rotational Doppler shift. Let us denote the values of the transmitting OAM beams as *l* and −*l*, the frequency shifts are Δ*f*_*l*_ and Δ*f*−_*l*_, using Eq. (2), the angular frequency of a spinning propeller can be calculated by

4 $${\rm{\Omega }}=\frac{2\pi ({\rm{\Delta }}{f}_{l}-{\rm{\Delta }}{f}_{-l})}{2l}$$

**Figure 8:**
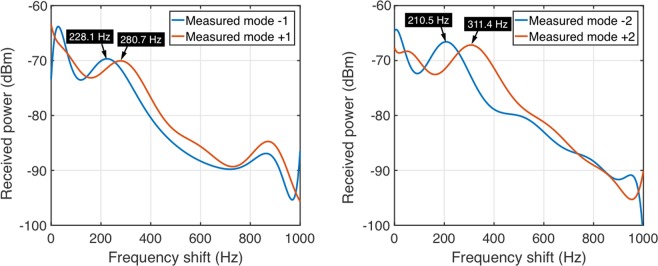
Measured frequency shifts of two opposite values of OAM beams reflected by a spinning helicoidal three-blades propeller. (**a**) Frequency shifts of *l* = −1 and *l* = + 1 OAM beams are 228.1 Hz and 280.7 Hz, respectively, and the frequency shift difference is 52.6 Hz. (**b**) Frequency shifts of *l* = −2 and *l* = + 2 OAM beams are 210.5 Hz and 311.4 Hz, respectively, and the frequency shift difference is 100.9 Hz. The calculated spinning speed by both *l* = ± 1 and *l* = ± 2 OAM beams have a good agreement with the actual speed measured by a high-speed camera, the errors are less than 2.7%.

Three-blades propeller is widely used in our daily life, here we propose to use two OAM beams with opposite value to measure the spinning speed of a three-blades propeller. In this case, we experimentally demonstrate to use *l* = ± 1 and *l* = ± 2 OAM beams at 30 GHz to measure the spinning speed of a three-bladed propeller, the actual spinning speed of it is 25.83 × 2*π* rad/s which is measured by a high-speed camera. We measure the frequency shifts Δ*f*_*l*_ and Δ*f*-_*l*_ of the scattering signals, as shown in Fig. 8 (the peak in the received power corresponding to a frequency shift around 200–320 Hz is chosen for the calculation purpose). In order to eliminate the unwanted frequency shift, we measure the average frequency shift in 8 seconds. Then we can calculate the spinning speed of the propeller by Eq. (4). The calculated spinning speed by *l* = ± 1 and *l* = ± 2 OAM beams are 26.3 × 2*π* rad/s and 25.2 × 2*π* rad/s, respectively. The calculated results have a good agreement with the actual spinning speed of the propeller. The experiment detail is shown in the supporting information. From the experimental data we can also see that the Doppler shift is frequency dependent, the source at 30 GHz has a larger frequency shift than the one at 5.8 GHz, see Fig. 7(e), because this Doppler shift is related to the original frequency as show in Eq. (2). On the other hand, the rotational Doppler shift is frequency independent, which is only related to the angular frequency and OAM mode. The three-blades propeller under test has an elevation height of 42 mm. Ideally it can be considered as an *l* = + 8.4 at 30 GHz, frequency shift for *l* = + 1 and *l* = −1 incident waves calculated by Eq. (2) will be 299 Hz and 350 Hz, respectively. It should be noted that the propeller used is not a perfect OAM generator especially in high order OAM mode, thus there is a frequency shift difference between the measured results and calculated results by Eq. (2), same phenomenon for *l* = + 2 and *l* = −2 incident waves. However, using two opposite values OAM beams can measure the difference of the rotational Doppler shift then calculate the spinning speed of the propeller correctly.

## Discussion

In this paper, we have used a spinning metasurface to detect the rotational Doppler effect in a radar system. The proposed metasurfaces with a polarization symmetrical structure are capable of generating a vortex beam while spinning. In our experiment, the frequency shift can be controlled by the spinning speed and the OAM mode of the metasurface. In addition, the spinning metasurface has the potential to be applied for the demonstration of Doppler radar deception or cloaking. By covering a moving object with the proposed spinning metasurface, one shall be able to manipulate the Doppler shift by generating a rotational Doppler shift component. The result of this arbitrary additional frequency shift would deceive a Doppler radar to detect the object for a given speed, despite the fact that it is actually moving at another different speed. Particularly, when the spinning metasurface generates a frequency shift opposite to that of the Doppler shift, the object would appear at rest to the Doppler radar. Moreover, an electronic control of static OAM metasurface can be designed to control the reflection phase of each unit then to mimic the spinning motion and generate a frequency shift for Doppler radar deception or cloaking.

Rotational Doppler effect has been used to measure the angular frequency of a planar surface. However, propellers that have a helicoidal structure instead of a planar structure are widely used in our daily life. A propeller consisting of three blades has the same structure as the helicoidal reflector, which had been proposed to generate vortex beam. Therefore, when a radar is used to measure the frequency shift of a spinning propeller it has not only rotational Doppler shift but also Doppler shift due to the rotational and translational motion of the propeller, respectively. In order to measure the spinning speed of a propeller without knowing its structure, we have used two opposite values of OAM beams illuminating to a propeller to measure the frequency shift difference. The measured results have a good agreement with the actually spinning speed of the propeller. This method may pave a new way to detect and measure the spinning speed of such propellers located on vehicles such as helicopters, drones, and submarines. The proposed method can be used to measure the spinning speed of a rotating target undergoes a translation motion or vibration.

## Method

The skew angles of different OAM beams are simulated using the commercially available multi-physics solver, COMSOL. All of the polarization-independent OAM metasurfaces are printed on a FR4 substrate and rotated by a motor. We measured the spectrograms via a radar operating at 5.8 GHz which is BumbleBee Radar by the Samraksh Company. The helicoidal reflector was fabricated by 3D printing and coated with metallic materials. For the spinning speed of propeller measurement, two opposite values OAM beams were used, each of OAM-carrying mm-wave beams at 30 GHz, which were generated with a specifically designed spiral phase plate. The frequency shifts were measured by a Rohde & Schwarz FSP spectrum analyzer. The actual speed of the metasurfaces, helicoidal reflector and propeller were measured by a high-speed camera.

## Supplementary information

Experimental Observation of Linear and Rotational Doppler Shifts from Several Designer Surfaces
