# Supplementary material for: Experimental Observation of Linear and Rotational Doppler Shifts from Several Designer Surfaces
Source: Sci Rep. 2019 Jun 20;9:8971. doi: 10.1038/s41598-019-45516-1 (PMC6586861; doi:10.1038/s41598-019-45516-1)
Supplement: Supplementary file 1 — Experimental Observation of Linear and Rotational Doppler Shifts from Several Designer Surfaces [file 41598_2019_45516_MOESM1_ESM.docx]

Experimental Observation of Linear and Rotational Doppler Shifts from Several Designer Surfaces

Baiyang Liu^1,2^, Hongchen Chu^1,3^, Henry Giddens^1^, Ronglin Li^2,*^ and Yang Hao^1,*^

^1^ Queen Mary University of London, School of Electronics Engineering and Computer Science, London E1 4NS, UK.

^2^ South China University of Technology, School of Electronics and Information Engineering, Guangzhou 510640, China.

^3^ Soochow University, College of Physics, Optoelectronics and Energy and Collaborative Innovation Center of Suzhou Nano Science and Technology, Suzhou 215006, China.

Y. H. (email: [y.hao@qmul.ac.uk](mailto:y.hao@qmul.ac.uk))
R. L. (email: [lirl@scut.edu.cn](mailto:lirl@scut.edu.cn))

For the metasurfaces using for rotational Doppler effect observation, they are polarization independent which can generate OAM beam while the metasurfaces are spinning. The design details are shown in Fig. 1. The proposed metasurfaces are modeled and simulated by CST 2016.


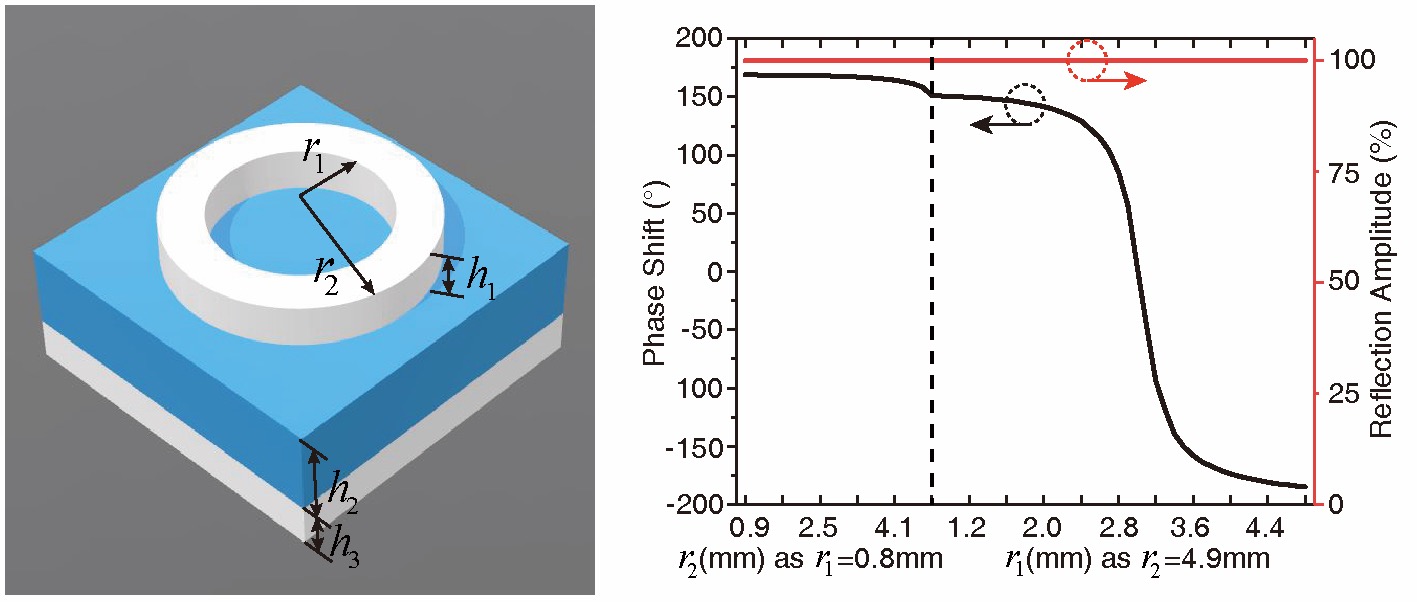


(a) (b)


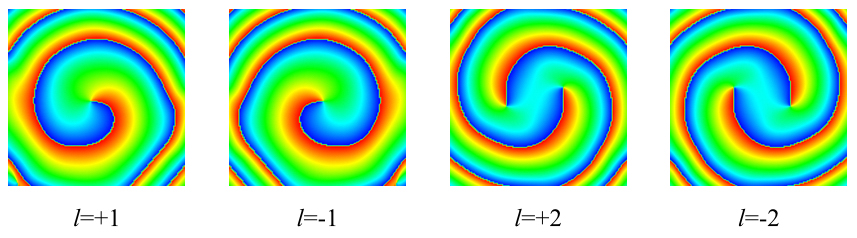


(c)


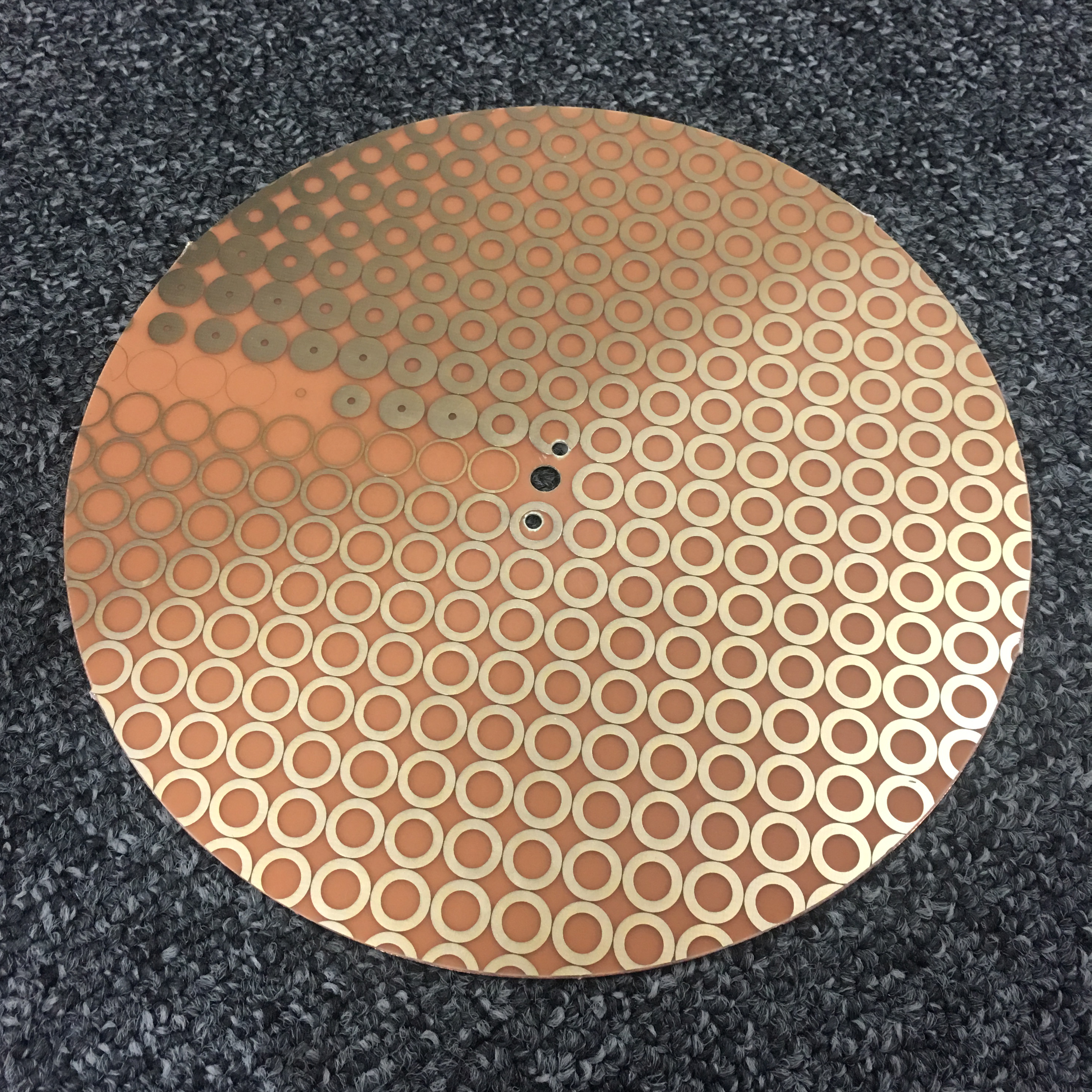


(d)

Fig. 1 (a) The schematic of the meta-atom with variables (outer radius r_2_, and inner radius r_1_) and constants (the upper metal ring thickness h_1_, the spacer thickness h_2_, the lower metal plane thickness h_3_). (b) Phase shift (black line) and normalized amplitude (red line) of reflection wave versus the outer and inner radii of the annular ring-shaped antenna at 5.8 GHz. (c) Simulated near-field phase of the metasurfaces with different OAM modes. (d) The prototype of the fabricated *l*=+1 metasurface, the holes on the prototype are used for mounting the metasurface on a motor.

The radar used in our experiments is BumbleBee Radar from The Samraksh Company, for more information of the radar, please search <https://samraksh.com/index.php/products/sensors/32-product-pages/products-sensors/71-bumblebee-radar>.

The *l*=+1 helicoidal reflector is designed at 5.8 GHz which is matched with the radar system. The helicoidal reflector is modeled and simulated by CST 2016, and is fabricated by Objet30 3D printer and spraying mental (RS Silver Coated Copper Screening Compound), as shown in Fig. 2. The elevation height of the helicoidal reflector is 25.85 mm which is λ/2 at 5.8 GHz.


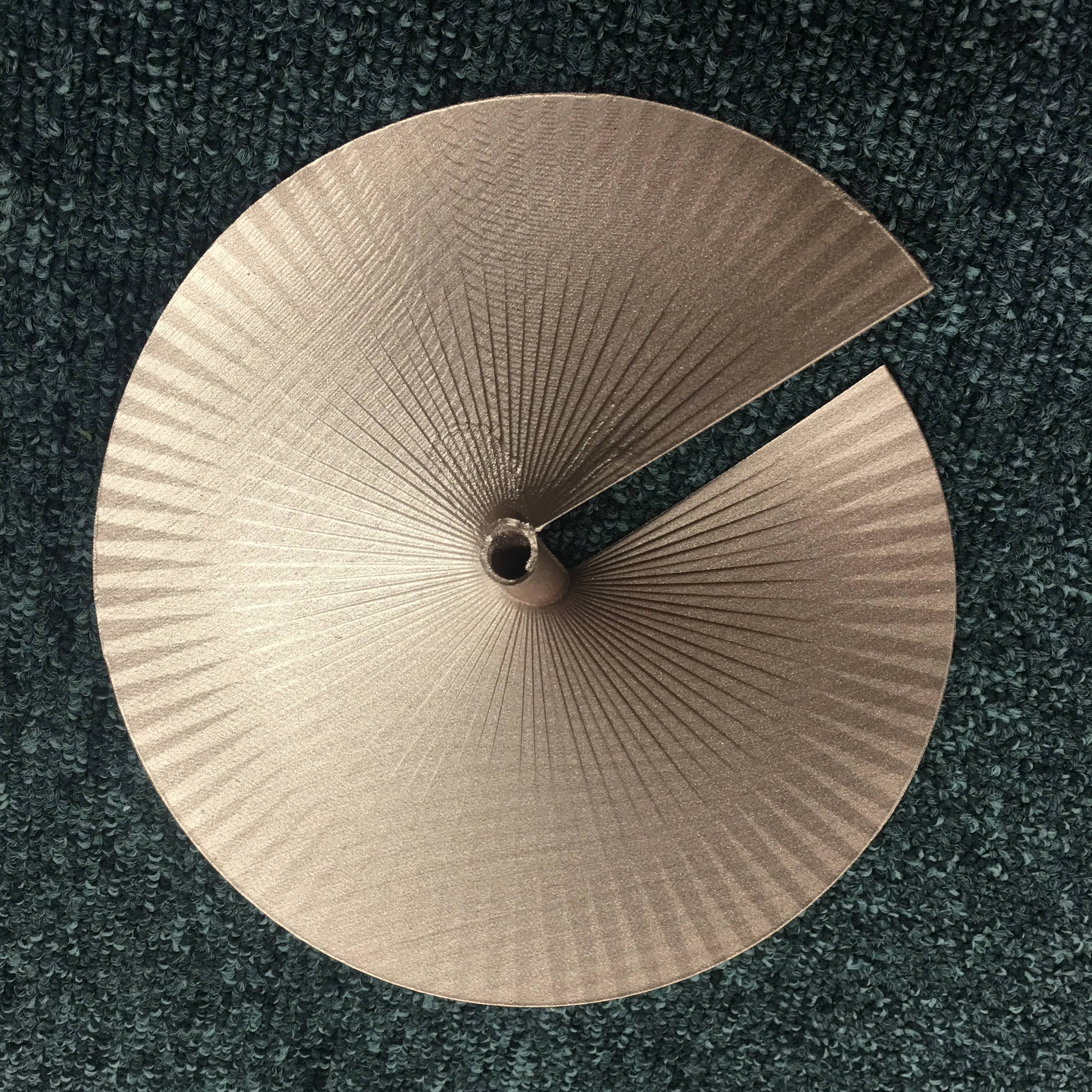

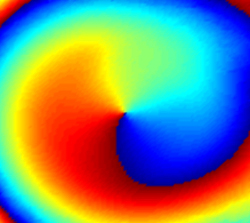


(a) (b)


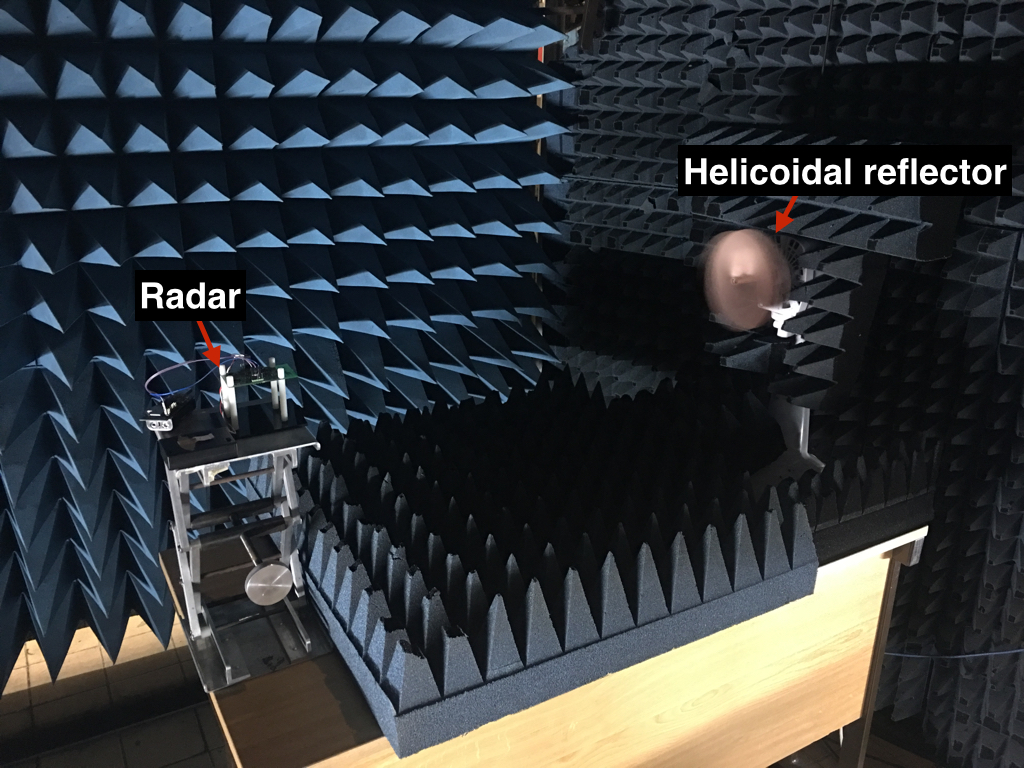


(c)

Fig. 2 The *l*=+1 helicoidal reflector for multiple Doppler effects observation. (a) Fabricated model; (b) Simulated near-field phase; (c) Spectrogram measurement of the spinning *l*=+1 helicoidal reflector.

All the SPPs to detect the spinning speed of a helicoidal three blades propeller are created by the Objet30 3D printer using the DurusWhite RGD430 Polypropylene-like material. The refractive index is 1.638. The designed SPPs’ models are shown in Fig. 3, the elevation height of each step is 15.16 mm, and the measure near-field helical phase are shown in Fig. 4.


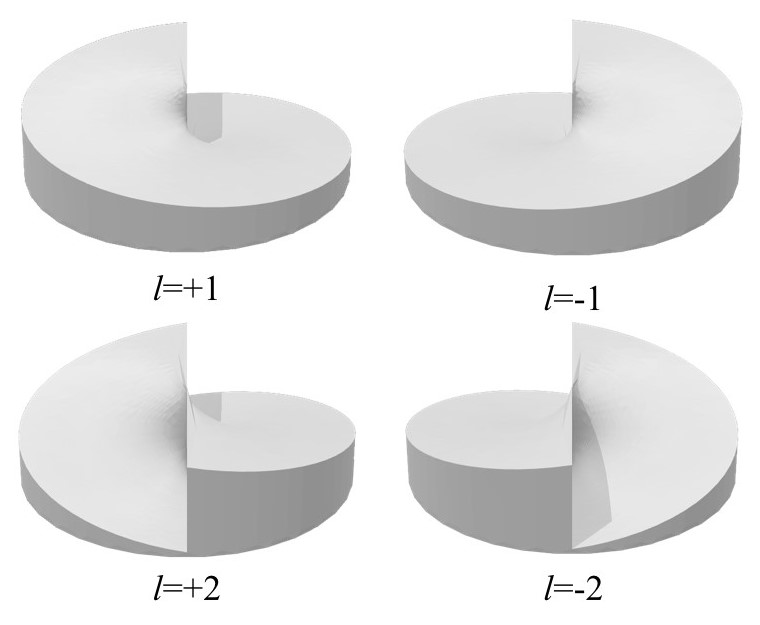

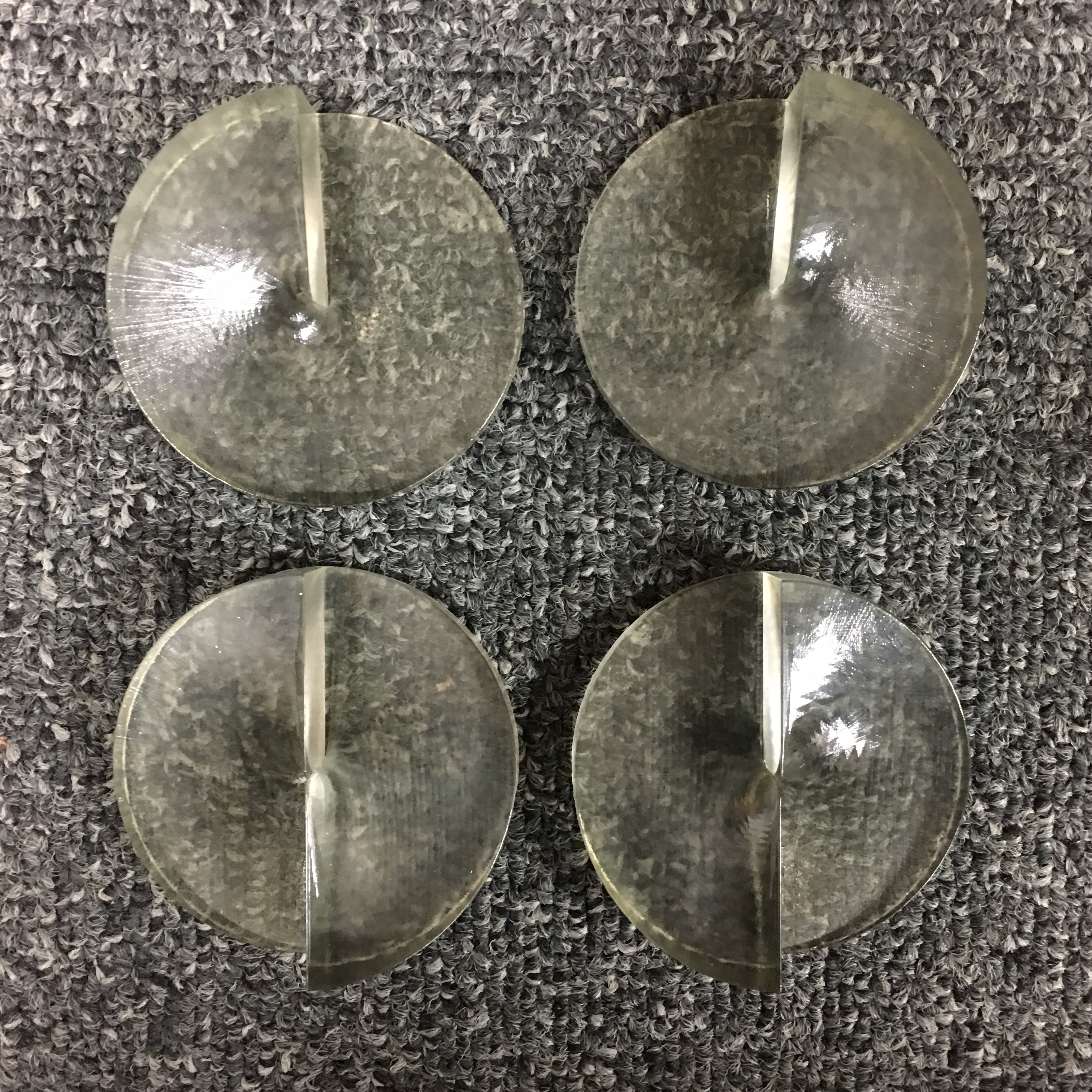


1. (b)

Fig. 3. Models of designed SPPs for OAM beam generation, (a) Simulation models; (b) Fabricated models.


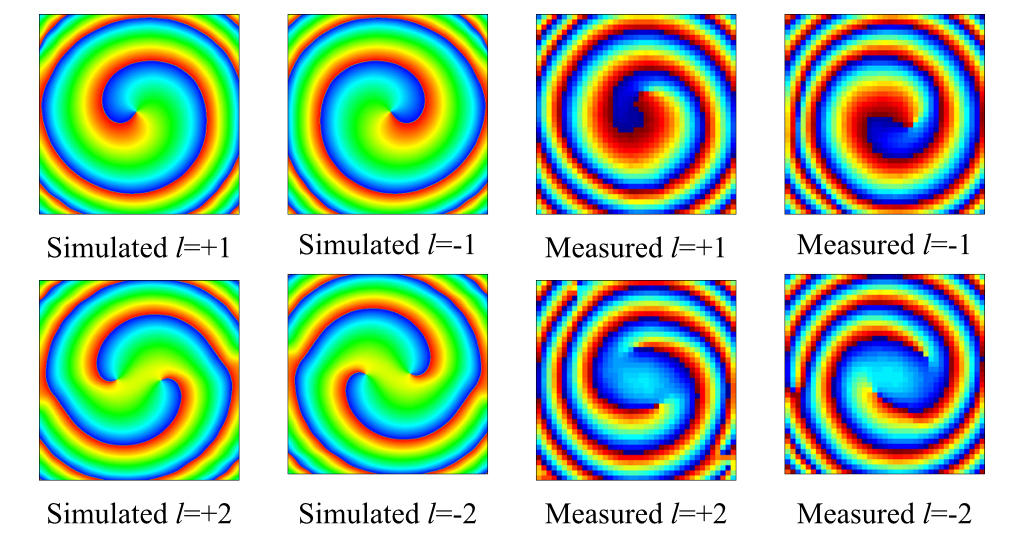


Fig. 4. Simulated and measured near-field helical phase of OAM beams generated by the proposed SPPs.

Fig. 5 is the experiment setup of the spinning speed measurement of a helicoidal three blades propeller. The transmitting antenna is connected to a signal generator (Agilent 83640L) generating 30 GHz sine wave, and the receiving antenna receives the scattering OAM waves by the spinning helicoidal three blades propeller, and then is connected to the spectrum analyzer (Rohde & Schwarz FSP spectrum analyzer 9 KHz to 40 GHz) to measure the average frequency shift in 8 seconds. *l*=±1 and *l*=±2 are used the generated opposite values of OAM beam to measure the difference of the rotational Doppler effect then calculated the spinning speed of the propeller.


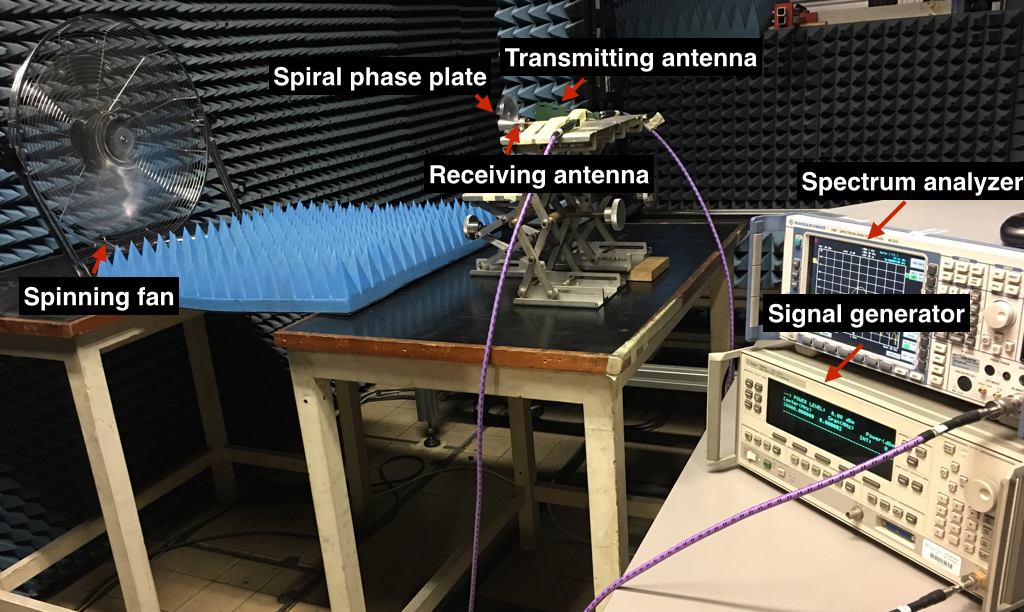


Fig.5 Experiment setup of the spinning speed measurement of a helicoidal three blades propeller.
